# Selective Breeding and Trait Improvement of Insect Natural Enemies for Ecologically Robust Biological Control

**DOI:** 10.3390/insects17070734

**Published:** 2026-07-17

**Authors:** Xu Chen, Jie Wang, Yong-Ming Chen, Lian-Sheng Zang, Su Wang

**Affiliations:** 1 Key Laboratory of Natural Enemies Insects, Ministry of Agriculture and Rural Affairs, Institute of Plant Protection, Beijing Academy of Agriculture and Forestry Sciences, Beijing 100097, China; chen_xuu@163.com; 2 State Key Laboratory of Green Pesticides, Guizhou University, Guiyang 550025, China; angusbio@126.com (Y.-M.C.); lszang@gzu.edu.cn (L.-S.Z.); 3 College of Life and Environmental Sciences, Hangzhou Normal University, Hangzhou 311121, China; wj_insect@126.com

**Keywords:** biological control, natural enemies, selective breeding, artificial selection, genetic improvement, quality control

## Abstract

Insect natural enemies are central agents in biological-control programs. Their pest-suppression performance depends not only on release strategy, but also on traits shaped by long-term laboratory rearing and selection. Conventional mass-rearing systems usually evaluate insect natural enemies using production-oriented indicators, such as survival, fecundity, emergence rate, sex ratio and production cost. However, continuous mass rearing can drive laboratory adaptation, genetic drift, inbreeding, shifts in host or prey use, and reduced field performance. In this review, we summarize key target traits for insect natural enemy improvement, including production efficiency, host or prey use, parasitism, predation, nonreproductive host killing, stress tolerance, pesticide compatibility, dispersal, retention and field efficacy. We further discuss how recurrent artificial selection, hybridization, population genetics, and genomics approaches can be integrated to improve insect natural enemies while maintaining genetic quality. Finally, we discuss the links among trait screening, selection design, quality control, storage and transport, and field-performance validation in the development and application of selected lines or improved strains. Overall, this review highlights practical directions for developing stable and field-proven natural enemy lines. Beyond agriculture, this perspective may also support ecological design in urban landscapes through natural enemy conservation and reduced pesticide use.

## Simple Summary

Natural enemy insects, such as tiny parasitoid wasps, ladybirds and lacewings, are widely used to control crop pests and reduce reliance on chemical pesticides. However, insects that are reared in the laboratory for many generations may change over time, and some may lose traits needed for effective pest control in the field. This review explains which traits are important when improving beneficial insects, such as survival, reproduction, pest-killing ability, tolerance to stress and performance after release. It also discusses how careful rearing, selection and modern genetic tools can help produce more reliable insects for safer and more sustainable pest management.

## 1. Introduction

Insect natural enemies, including parasitoid wasps, predatory bugs, lacewings, ladybird beetles and other entomophagous insects, are important components of biological control. Biological control can be broadly divided into classical, conservation and augmentative approaches, which differ in their objectives, operational procedures and expected ecological outcomes. Classical biological control generally involves the exploration, importation, evaluation and release of host-specific natural enemies to establish self-sustaining populations against invasive pests. Conservation biological control focuses on habitat or landscape management to enhance the activity and persistence of existing natural enemy communities. In contrast, augmentative biological control relies on the mass production, storage, transport and periodic release of natural enemies to suppress pest populations in managed agricultural systems. In several regions, applied biological control has moved from laboratory assays to field-scale programs supported by insect rearing techniques, bio-factories and standardized production chains [1,2,3]. This transition has been particularly visible in systems such as *Trichogramma* releases against lepidopteran pests of maize in many countries, *Tamarixia radiata* production for management of the Asian citrus psyllid, and egg parasitoid systems based on factitious hosts [4,5,6,7,8,9].

Despite these advances, the effectiveness of augmentative biological control remains uneven across inundative and seasonal inoculative release programs, particularly when natural enemies are deployed in open agricultural systems or in greenhouse crops where pollinators and other beneficial insects are also released. Commercially reared insects may perform well under controlled rearing conditions but fail to disperse appropriately, locate hosts or prey, tolerate field stress, remain in the crop, or suppress target pests after release [1,10]. This mismatch occurs because the traits that favor efficient production are not always the same traits that favor biological control. A colony that grows rapidly in the laboratory may have been unintentionally selected for high fecundity on a factitious host, tolerance of crowding, reduced dispersal, altered courtship behavior, or simplified host-searching responses, while losing genetic diversity or field-relevant performance [11,12,13,14]. In addition, poor post-release performance may result from pesticide residues and inappropriate release timing in conventional cropping systems, where insecticides or acaricides can reduce the survival and establishment of released parasitoids and predators [15,16,17,18].

Selective breeding and genetic improvement provide a more intentional approach to managing the evolutionary changes that occur during mass rearing. Instead of allowing long-term, multi-generation rearing in bio-factories to drive uncontrolled changes in colony traits, beneficial insect lines can be deliberately screened, selected and maintained for traits that directly improve their compatibility with augmentative biological-control programs [1,19]. This shift is important because mass-reared natural enemies are not merely biological products; they are evolving populations whose performance depends on the balance between production efficiency, genetic quality and field function [12,13]. In practice, useful variation may already exist among geographic populations, host-associated populations, laboratory strains or commercial stocks, providing raw material for selection before advanced genetic tools are considered [20,21,22,23,24]. Recent studies have selected or evaluated traits related to pesticide resistance, cold tolerance, starvation tolerance, nonreproductive host killing, zoophagy, body size, pollen feeding, wing truncation, reduced flight ability and field retention, indicating that insect natural enemies can respond to directional selection in traits relevant to augmentative biological control [2,17,25,26,27,28,29,30,31,32,33,34,35].

However, a trait-oriented breeding perspective also requires caution. Improvement of one trait can generate costs in other traits or in different ecological contexts. For example, selection for pesticide resistance may increase compatibility with pesticide-based integrated pest management (IPM) programs, but it may also affect fitness, prey consumption, reproductive output or long-term genetic stability [17,29,32]. Selection for reduced flight may increase crop residence but reduce dispersal, escape ability or survival under some conditions [25,34]. Selection for zoophagy, here referring to increased feeding on target animal prey rather than plant tissues or conspecifics, may improve pest suppression by omnivorous predators, but it may also increase risks of cannibalism, intraguild predation or plant and fruit damage when prey availability is low or when predators shift between prey feeding and phytophagy [36]. Therefore, selected lines and improved strains should not be evaluated only through the selected trait; they should be tested through multi-trait quality assessment and field-performance validation [37,38,39,40].

**Figure 1:**
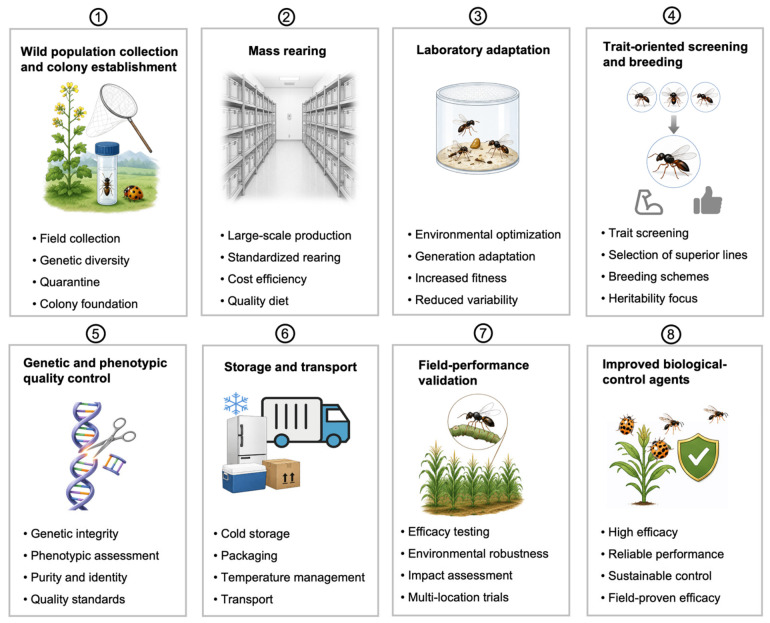
Conceptual framework for rearing-associated adaptation and selective rearing of insect natural enemies. The framework links wild population collection, colony establishment, mass rearing, laboratory adaptation, trait-oriented screening and breeding, genetic and phenotypic quality control, storage and transport, field-performance validation, and the development of improved biological-control agents. Feedback loops indicate that field performance should inform subsequent breeding targets and quality-control (QC) decisions.

Building on this background, the present review focuses on trait-oriented improvement of insect natural enemies rather than treating mass rearing, rearing-associated adaptation and quality control as isolated processes. It places particular emphasis on the breeding value of biological-control-related traits, the evidence for selected lines or improved strains, and the ways in which artificial selection and genetic or genomic tools can be integrated into a practical improvement pipeline. Specifically, this review addresses three linked questions: which traits are most relevant for selection in insect natural enemies; what evidence exists that selected lines or improved strains show enhanced biological-control performance; and how adaptation during mass rearing can be combined with genetic, genomic and phenotypic quality management to produce natural enemies that are not only efficiently mass-reared but also biologically effective after release (Figure 1). By examining these aspects, this review reframes mass-reared insect natural enemies as evolving biological-control populations rather than static commercial products. A structured literature search was conducted to identify studies on rearing-associated adaptation, selective breeding, and trait improvement of insect natural enemies used in biological control. The Web of Science Core Collection was used as the primary database, with keyword combinations including “biological control”, “natural enemies”, “mass rearing”, “selective breeding”, and “trait selection”. The initial search retrieved 966 records, which were subsequently screened in two stages based on titles and abstracts, followed by full-text evaluation. Studies were included if they focused on parasitoids or predators used in biological control and reported evidence of trait variation, selection response, or performance changes during rearing or breeding processes. Studies exclusively addressing chemical control, plant resistance, or non-insect biological agents were excluded. The final dataset consisted of representative studies used for qualitative synthesis in this review.

## 2. Rearing-Associated Adaptation During Mass Rearing: Sources of Variation and Constraints

Rearing-associated adaptation of insect natural enemies can be defined as the process through which wild or field-collected populations become adapted to artificial rearing conditions. It begins with collection and colony establishment, continues through adaptation to laboratory temperature, humidity, density, host or prey supply, photoperiod and diet, and may be reinforced by routine handling, storage and transport [3,41].

### 2.1. Colony Establishment and Early Rearing-Associated Adaptation

The initial step in the genetic improvement of natural enemies is typically not direct selective breeding, but rather colony establishment. The quality of this establishment directly determines the abundance of initial genetic variation available for subsequent screening and breeding. In practice, however, small founder populations (the founder effect), unbalanced sex ratios, repeated genetic bottlenecks, and prolonged closed rearing can drastically reduce the effective population size (*N_e_*) and exacerbate inbreeding depression. This issue is particularly lethal in hymenopteran parasitoids with single-locus complementary sex determination (sl-CSD), as high levels of inbreeding lead to the production of sterile diploid males, triggering rapid colony collapse [42,43]. Concurrently, comparative studies between wild and commercial or laboratory populations confirm that the life-history traits of natural enemies diverge significantly during indoor rearing. This unintentional laboratory adaptation implies that laboratory colonies have often lost their tolerance to extreme environments or complex host-searching behaviors; therefore, they can never be simply treated as “neutral representatives” of natural field populations [13,21].

To achieve efficient, trait-oriented breeding, colony establishment must be strategically designed as the construction of a genetic resource. Rather than maintaining a single colony with an extremely narrow genetic background, modern biocontrol programs should adopt a more systematic strategy: widely collecting multiple wild populations across different climatic zones and host ecotypes, documenting their geographic and ecological origins in detail, maintaining independent backup lines, and periodically introducing wild individuals for rejuvenation to sustain colony vigor [44]. Furthermore, the phenotypes of laboratory lines and natural field populations should be periodically benchmarked against one another [12,13]. This colony-establishment strategy provides baseline information on source populations and preserves selectable variation for subsequent screening and quality control. During long-term, multi-generation rearing, reared colonies should be periodically evaluated for genetic diversity, fitness-related traits, symbiont or microbiota status such as *Wolbachia* infection, and release-relevant performance before they are used in inundative or augmentative biological-control programs [45,46].

### 2.2. Factitious Hosts, Alternative Hosts and Artificial Diets as Selective Environments

Laboratory adaptation differs from deliberate selective breeding. Laboratory adaptation is often an unplanned response to the artificial rearing environment, whereas selective breeding imposes intentional selection on specified traits. Specifically, empirical evidence highlights several key dimensions of trait modifications driven by the artificial rearing environment and the use of specific factitious hosts.

Factitious hosts, such as the rice moth *Corcyra cephalonica*, provide a practical basis for cost-effective, year-round mass production of biological-control agents, but this rearing history can also reshape parasitoid phenotypes [47]. First, morphological and physiological traits are highly responsive to host substitution. For example, rearing history drives physiological adaptations, such as significant shifts in digestive enzymatic activity and life-history parameters in *Habrobracon hebetor* [48]. Furthermore, the specific factitious host species directly limits or enhances offspring body size and adult longevity, as demonstrated when comparing *Trichogramma ostriniae* reared on *Antheraea pernyi* versus *C. cephalonica* [49]. Second, reproductive and behavioral parameters frequently undergo significant modifications. The properties and preparation of factitious host eggs can directly alter the oviposition preference and initial host acceptance of eupelmid parasitoids [50]. Most critically, continuous laboratory rearing over successive generations triggers a marked decline in overall quality; for instance, *Trichogramma brassicae* continuously maintained on *Ephestia kuehniella* exhibits a severe degradation in lifetime fecundity, reproductive rates, and finite parasitism capacity after 20 generations [44]. QC tests and bioassays after successive generations on factitious hosts are needed to evaluate host acceptance, emergence, fecundity, sex ratio, longevity and parasitism capacity before release.

Predatory insects experience similar phenotypic shifts and selective pressures when maintained on artificial diets designed to reduce rearing costs. Empirical evidence across multiple taxa—including *Harmonia axyridis*, *Cryptolaemus montrouzieri*, *Chrysoperla externa*, *Orius majusculus*, and *Propylea japonica*—demonstrates that dietary macronutrient composition critically influences immature survival, developmental duration, adult body mass, and lifetime fecundity [51,52,53,54,55,56]. While artificial diets are primarily evaluated for their immediate capacity to replace expensive factitious prey and sustain continuous generations (e.g., sustaining *C. externa* for up to seven generations), they frequently induce severe physiological trade-offs. For instance, sub-optimal artificial diets can lead to delayed development, reduced adult size, and drastically impaired reproductive output—even causing a complete cessation of oviposition in some adult ladybirds unless natural prey is reintroduced. Therefore, beyond mere production feasibility, artificial diets must be rigorously assessed for how they shape the long-term trait profile of the colony. A dietary regime that lowers economic costs but inadvertently degrades reproductive quality, physical fitness, or subsequent hunting performance is fundamentally incompatible with the development of high-efficacy biological-control agents.

Because rearing substrates can impose selection, factitious hosts and artificial diets should not be considered purely technical inputs. They can either constrain or enable improvement. For example, a large factitious host may produce larger parasitoids or improve some production traits, but the resulting line must still be tested on the target pest and under field-relevant conditions [49,50,57]. Similarly, nutritional enhancement of predator diets can increase survival or production, but the link between diet-derived laboratory performance and field predation must be demonstrated [53,58,59].

### 2.3. Continuous Rearing, Genetic Drift and Reduced Biocontrol Efficacy

Continuous rearing may lead to progressive changes in natural enemy quality. In *Trichogramma* and other parasitoids, long-term laboratory maintenance has been associated with altered fitness-related traits, changes in courtship signals, modified host acceptance and reduced performance under some conditions [11,14,39,44]. Genomic evidence from *Aphidius gifuensis* further supports this concern: long-term captive rearing was associated with reduced genetic diversity, whereas wild populations retained genetic variation partly through natural selection linked to host plants and climatic conditions [12]. Similar concerns apply to predatory insects. Commercial or laboratory colonies of predators such as *O. majusculus* and *H. axyridis* may show changes in genetic diversity [60], diet-related fitness, flight ability, residence time, survival or fecundity under artificial rearing or selection regimes [13,25,35,52,59]. These findings indicate that both parasitoid and predator colonies can change during mass rearing, and such changes may reflect laboratory adaptation, inbreeding, genetic drift or selection imposed by simplified rearing protocols.

However, not all rearing-associated changes necessarily reduce production value. Some changes induced by long-term rearing may improve handling tolerance, development on factitious hosts or synchronization with production schedules. These changes should not be interpreted as true domestication, but as colony-level responses to artificial rearing environments. The key issue is whether such production-related advantages are maintained without reducing field-relevant performance after release. Selected traits, including pesticide resistance, may also carry fitness costs or become unstable when released into environments where selection pressures are absent or variable. Therefore, breeding and quality-control programs should evaluate production traits, fitness traits, genetic stability and field-relevant behaviors over generations, rather than relying on a single laboratory indicator such as emergence, fecundity or parasitism in small arenas [37,38,39,40,61].

## 3. Target Traits and Trade-Offs in Selective Breeding of Insect Natural Enemies

Selective breeding, in contrast, requires explicit breeding objectives, defined base populations, phenotyping protocols, selected and control lines, selection intensity, repeated generations, and evaluation of correlated responses. In the context of insect natural enemies, genetic improvement should not be equated with the immediate commercial deployment of transgenic or gene-edited strains. Rather, it includes the use of natural variation, artificial selection, line crossing, population genetics, genomics, transcriptomics, proteomics and microbiome-associated information to guide improvement and maintain quality [19,58,62,63,64,65]. This distinction is important because many current programs can adopt genetic monitoring or genomic resources without moving immediately to genetically modified organisms. Moreover, the deliberate release of transgenic or gene-edited entomophagous insects would require rigorous ecological risk assessment and regulatory approval, including evaluation of establishment, dispersal, persistence, host or prey range, non-target effects and gene flow.

Trait selection should begin with a clear definition of the biological-control problem (Table 1). For instance, a natural enemy used in greenhouse thrips control may need different traits from a parasitoid released into maize fields, a predator released against aphids in orchards, or a parasitoid shipped as pupae for augmentative release. The breeding objective should therefore combine production feasibility, release logistics, crop environment, pest biology and compatibility with IPM programs [10,19,66].

A useful distinction is between traits that improve the economics of production and traits that improve pest suppression. Production traits include survival on artificial diets, development rate, fecundity, adult emergence, sex ratio, tolerance of crowding, storage survival, production cost and reduced cannibalism under mass-rearing conditions [61,67,68,69,70]. Pest-suppression traits include parasitism, predation, functional response, host killing, prey switching, host or prey location, dispersal, retention and field efficacy [31,38,71,72]. Improvement programs must avoid selecting exclusively for production traits if these compromise pest suppression.

Stress tolerance traits are increasingly important because natural enemies often encounter temperatures, humidity levels, starvation periods, transport and mechanical or manual handling during release, which can differ substantially from standard laboratory rearing conditions [73]. Strain-specific differences in temperature-dependent reproduction and survival have been reported in *Trichogramma*, and breeding for cold tolerance has been explored in *Orius laevigatus* [20,24,26]. Diapause expression and its plasticity have been shown to strongly affect establishment and efficacy in parasitoid systems, highlighting its relevance for release timing and population persistence [74]. Starvation resistance has also been selected in *Pachycrepoideus vindemmiae*, showing that stress-related traits can respond to artificial selection and may influence broader biological-control performance [27,75].

Pesticide compatibility is another major target for the improvement of insect natural enemies. In many augmentative biological-control programs, natural enemies are released into crops where insecticides or acaricides are still used, either because pest pressure is high, multiple pest species occur simultaneously, or growers rely on chemical products as part of integrated pest management. Under these conditions, selected lines that can tolerate selective or commonly used pesticides may improve the temporal and operational compatibility between natural enemy releases and pesticide applications. *Orius laevigatus* provides one of the clearest examples of this approach. Artificial selection has been used to increase tolerance to insecticides such as emamectin benzoate, pyrethroids, and spinosad, indicating that pesticide tolerance can be enhanced under controlled selection regimes and may contribute to improved compatibility with IPM-based crop protection systems [17,29,32]. More broadly, the identification of pesticide tolerance as a target trait is consistent with recent discussions on genetic improvement of predatory arthropods in protected crops, where compatibility with existing pest management programs is considered a key requirement for commercial success [66]. However, pesticide resistance should not be evaluated in isolation. Resistant lines must be subjected to comprehensive QC assessments, including reproduction, prey consumption, survival, behavior, and population persistence, because selection for resistance may be associated with trade-offs that influence overall biological-control performance.

Dispersal and retention traits deserve special attention. Natural enemies that disperse too rapidly may leave the crop before suppressing pests, whereas natural enemies with reduced dispersal may remain longer but fail to colonize patches or escape unfavorable conditions. Artificial selection for reduced flight ability and genetic analysis of wing truncation in *H. axyridis* show that retention traits can be modified, but also reveal possible survival and fitness costs [25,34,35]. Thus, retention should be treated as a context-dependent trait rather than a universal breeding objective.

Finally, nutrition-associated and plant-associated traits are relevant for polyphagous predators. Selection for better pollen-feeding fitness in *O. laevigatus* and zoophagy in *Dicyphus hesperus* illustrates that breeding can target performance under prey scarcity and plant-mediated contexts [30,36]. These traits are valuable in protected crops where predators rely partly on pollen or phytophagy before pest outbreaks. At the same time, selection for stronger zoophagy or phytophagy may increase the risk of plant damage, requiring evaluation of both pest suppression and crop injury.

**Table 1:** Trait categories that can be targeted in selective breeding and genetic improvement of insect natural enemies.

Trait Category	Representative Traits	Main Biological-Control Value	References
Production and colonization	Fecundity, survival, emergence, development time, sex ratio, adaptation to factitious hosts or diets	Determines whether the line can be produced economically and consistently	[61,67,68,69,70]
Host/prey use and pest suppression	Host acceptance, parasitism, predation, functional response, nonreproductive host killing	Determines direct pest mortality after release	[22,31,38,72,76]
Stress tolerance	Heat, cold, starvation, desiccation and storage tolerance	Improves survival during transport, release and adverse field conditions	[20,24,26,27,77,78,79]
Pesticide compatibility	Resistance or tolerance to selective insecticides and sublethal exposures	Allows integration with pesticide-based IPM programs	[17,29,32]
Dispersal and retention	Flight ability, wing form, crop residence, patch leaving and density-dependent dispersal	Affects spatial distribution and persistence after release	[25,34,35,80,81]
Nutritional and plant-associated traits	Pollen feeding, artificial diet performance, zoophagy, plant feeding	Supports predator persistence before or between pest outbreaks	[30,36,52,58,59]

## 4. Selected Lines and Improved Strains: Current Evidence Across Natural Enemy Groups

### 4.1. Predatory Bugs as Model Systems for Trait-Oriented Improvement

Predatory bugs have become prominent models for trait-oriented improvement because they are commercially relevant, can be reared in the laboratory and show variation in pesticide tolerance, nutrition-related fitness, body size, predation and plant-associated traits. *Orius laevigatus* represents a well-established example in this context, where artificial selection has been used to enhance tolerance to pyrethroids, spinosad, and emamectin benzoate, with the aim of improving compatibility between predator releases and pesticide-based IPM programs [17,29,32]. Importantly, while these studies demonstrate the feasibility of selecting for pesticide tolerance, they also highlight the necessity of evaluating potential trade-offs in prey consumption, fecundity, and population persistence when assessing the biological-control value of selected lines.

*Orius laevigatus* has also been used to study selection for traits other than pesticide resistance. Selection for larger body size and better fitness when feeding on pollen indicates that production and nutritional traits may be improved in ways that support predator establishment and performance in protected crops [30,33]. More recently, breeding cold-tolerant lines has addressed the need for predators that can tolerate lower temperatures, potentially expanding the temporal and geographic windows for release [26]. Together, these studies show that a single natural enemy species can be improved along several trait axes, but they also raise the question of multi-trait balance.

*Dicyphus hesperus* provides a complementary example in which artificial selection on foraging behavior generates more zoophagous lines with improved biological-control efficacy in greenhouse tomato systems. Highly zoophagous lines achieve rapid and sustained suppression of *Bemisia tabaci* populations while causing lower levels of fruit damage, whereas less zoophagous lines show delayed pest suppression and higher crop injury, indicating that variation in feeding behavior strongly affects both pest control efficiency and crop safety [36]. Importantly, selected lines remain compatible with Encarsia formosa, with minimal evidence of interference, highlighting their potential suitability for integration into multi-agent biological-control programs.

### 4.2. Parasitoids Selected for Stress Tolerance and Host Killing

Parasitoids offer several promising targets for artificial selection, including host location, host acceptance, clutch size, sex ratio, stress tolerance and nonreproductive host killing. *P. vindemmiae* is an informative example because selection has been applied to starvation resistance and nonreproductive host killing [27,31]. The starvation-resistant lines show that a stress-related trait can be improved and may influence multiple biological-control traits. Selection for nonreproductive host killing is especially important because parasitoids can suppress pests not only by producing offspring but also by killing hosts without successful parasitoid emergence [31].

The concept of nonreproductive host killing broadens the definition of parasitoid performance. In many applied programs, parasitism rate alone is an incomplete measure because hosts may be killed through host feeding, stinging, probing, physiological disruption or unsuccessful oviposition. Breeding programs should therefore consider total host mortality, parasitoid emergence, offspring quality and sex ratio as distinct but related outcomes. A line that increases host mortality without maintaining parasitoid reproduction may be useful in inundative release but less useful for inoculative establishment.

Egg parasitoids such as *Trichogramma* and *Telenomus* remain central to mass-rearing and QC research, even though fewer studies have implemented formal artificial selection. Variation among strains, host suitability, temperature-dependent traits, diapause-related traits relevant to storage and functional responses provide raw material for future breeding [20,22,24]. Successive rearing of *Telenomus remus* on *S. litura* eggs and QC studies of *T*. *brassicae* and *T. remus* show that parasitoid performance can change during production and must be monitored over generations [39,40,82,83].

### 4.3. Ladybird Beetles, Lacewings and Other Predators

Ladybird beetles and lacewings have long been used in biological-control research and provide useful examples of diet adaptation, dispersal modification and artificial diet development. *Harmonia axyridis* has been studied for nutritional fitness traits, artificial diet improvement [60] and reduced flight ability [25,34,35,53,59]. Artificial selection for reduced flight ability increased residence time in some contexts, but survival and other performance costs must be considered [25,35]. This example is valuable because it connects a concrete behavioral trait to post-release retention, a frequent weakness of augmentative releases.

Lacewings and coccinellids have also been studied through diet development and mass-rearing optimization. Artificial diets for *C. externa* and diet-based improvements for *H. axyridis* and *C. montrouzieri* support the idea that nutritional traits can be manipulated before formal selection begins [51,53,54,55,84]. However, diet performance alone should not be treated as an improved strain. To qualify as trait improvement, the diet-adapted or selected line should be compared with a control line for relevant field traits such as prey consumption, dispersal, reproduction and survival.

Collectively, the empirical evidence across predatory bugs, parasitoids, and other predators demonstrates that natural enemies can be successfully selected for specific targets, such as pesticide compatibility, stress tolerance, and field retention (Table 2). However, a unifying theme across all these model taxa is the inevitability of physiological and ecological trade-offs. Whether evaluating the risks of plant damage in polyphagous bugs, the balance between total host mortality and successful emergence in parasitoids, or the survival costs associated with reduced flight in ladybirds, enhancing a single trait often incurs hidden consequences. Therefore, the successful development of improved strains cannot rely on a single laboratory metric. It demands a comprehensive, multi-trait evaluation framework that rigorously monitors overall ecological fitness, ensuring that specialized improvements do not compromise the fundamental biocontrol efficacy.

## 5. Integrating Conventional Selection with Genetic and Genomic Tools

A practical improvement pipeline for insect natural enemies should begin with conventional steps: collecting diverse source populations, screening natural and laboratory variation, defining target traits, establishing control and selected lines, and estimating selection response over generations. These steps remain essential even when molecular tools are available, because genomic information is useful only when connected to reliable phenotypes and clear breeding objectives [19,66]. Genomics should be regarded as a support tool rather than a shortcut to improved strains, and its application requires caution because genomic-assisted improvement may generate unintended ecological risks if selected traits alter dispersal, host or prey use, competitive ability or interactions with non-target organisms.

Traditional selection can be used deliberately rather than passively. For example, colonies can be exposed to target hosts, factitious hosts, lower temperatures, pesticide regimes or artificial diets in controlled experimental-evolution designs. Such designs should include replicated selected lines and control lines, because otherwise it is difficult to separate true selection response from drift, founder effects or environmental noise. Long-term rearing studies in parasitoids already show that quality and behavior can shift over generations, which suggests that unplanned adaptation could be redirected into intentional improvement if properly monitored [11,14,39,44].

Hybridization and line crossing may provide another route to improvement. If different geographic or host-associated populations differ in heat tolerance, host acceptance or pesticide compatibility, crossing could combine desirable traits or generate heterosis. However, line crossing can also disrupt local adaptation or alter host range. For this reason, hybridization should be followed by host-specificity, fitness and field-performance tests before improved lines are released.

Genomic and transcriptomic resources are increasingly available for parasitoids and predators. Genome assembly of *Trichogramma dendrolimi*, transcriptomic analyses of thermal stress in *Trichogramma chilonis* and *Anisopteromalus calandrae*, and population genomic analyses of trophic interactions provide examples of how molecular data can inform trait interpretation and genetic monitoring [62,63,64,65]. Furthermore, transcriptomics can be practically integrated into selective breeding by identifying early biological markers (biomarkers) for phenotypic screening. Instead of relying solely on time-consuming phenotypic evaluations across multiple generations, breeders can utilize specific gene expression profiles—such as the rapid upregulation of heat-shock proteins or detoxification enzymes—to efficiently screen and validate stress-tolerant or pesticide-compatible lines at the molecular level [59,62,63,85]. Proteomics has also been used to examine responses of *Arma chinensis* to an improved artificial diet, linking nutritional improvement with underlying physiological mechanisms [58]. In the model parasitoid *Nasonia vitripennis*, genomic prediction based on genome-wide markers has been tested as a proof of principle for estimating the genetic merit of wing morphology traits, suggesting that genomic selection may be feasible for insect biocontrol agents but remains constrained by phenotyping methods, insect biology and genotyping costs [86]. Although gene-editing tools have been demonstrated in parasitoid model systems such as *N. vitripennis*, these examples remain primarily experimental and have not yet translated into commercially released genetically modified natural enemy strains [87].

Microbiome-based improvement represents a rapidly emerging, albeit cautious, frontier in the rearing-associated adaptation and augmentation of biological-control agents. Endosymbionts and host-associated microbiomes play profound roles in shaping the phenotypic plasticity and ecological adaptability of insect natural enemies. Specific symbiotic interactions can fundamentally modulate critical traits, including reproductive modes, sex ratio allocation, thermal and stress tolerance, host-use patterns, and even detoxification pathways for pesticide resistance. A classic paradigm is the *Wolbachia*-associated system, which heavily influences the reproductive biology (e.g., inducing thelytokous parthenogenesis) and directly dictates QC decisions in the commercial mass-rearing of *Trichogramma* wasps [88]. Furthermore, the paradigm of microbe-mediated augmentation is continuously expanding. Drawing inspiration from parallel advances in microbial biocontrol—such as the genetic engineering of fungal biocontrol agents to achieve greater efficacy against insect pests [89]—researchers are now exploring how synthetic biology and targeted microbiome engineering could be synergistically applied to the symbionts of insect natural enemies. However, despite its transformative potential, symbiont manipulation must be strictly integrated into the same rigorous evaluation framework as traditional genetic improvement. Before commercial deployment, it is imperative to comprehensively assess the cross-generational stability of the altered microbiome, the reliability of vertical versus horizontal transmission, the durability of trait effects, and, most importantly, the ecological safety and long-term field performance to prevent unintended disruptions in non-target ecosystems.

Therefore, the most realistic near-term strategy is not to replace conventional selection with genetic engineering, but to combine phenotypic selection with genetic and genomic monitoring. Genomics can help identify whether selected lines maintain sufficient diversity, whether commercial stocks are genetically depauperate, whether repeated bottlenecks occur, and whether selected phenotypes remain stable over production cycles [12,13,19,64]. To operationalize this in commercial bio-factories, the application of population genomics through low-cost Single-Nucleotide Polymorphism (SNP) arrays offers a highly scalable tool for routine genetic quality control. By genotyping mass-reared colonies using customized, species-specific SNP chips, producers can rapidly and economically monitor genetic drift, quantify inbreeding depression, and authenticate the genetic identity of improved strains across successive production cycles without relying exclusively on laborious biological assays. This integrated approach aligns with next-generation biological control while remaining compatible with current regulatory and commercial realities.

## 6. Quality Control, Genetic Stability and Field Validation of Improved Lines

QC is the bridge between breeding claims and biological-control value. A line should not be considered improved only because it performs better in a single laboratory assay. It should be evaluated against its base or control line using a multi-stage process that includes selected trait performance, correlated fitness traits, storage and transport tolerance, semi-field testing and field efficacy [2,37,38,40].

Routine phenotypic QC indicators include emergence, adult longevity, fecundity, sex ratio, flight ability, parasitism or predation, body size, host or prey acceptance, and response to storage [37,38,77,88]. For parasitoids, clutch size and sex ratio may be particularly important, especially in gregarious species or *Wolbachia*-infected thelytokous lines [88]. For predators, survival on diets, prey consumption, plant damage, dispersal and retention should be added to the assessment. Cold storage studies in *Microplitis manilae*, *Psyttalia incisi* and *T. dendrolimi* show that storage can affect reproductive and quality traits, and therefore should be incorporated into selected-line validation [77,78,79].

Genetic quality management should be added to conventional QC. Selected lines may be vulnerable to loss of diversity because artificial selection often reduces effective population size. Commercial stocks of biological-control agents can show reduced genetic diversity, and continuous biological-control application can impose natural selection while maintaining or reshaping genetic variation [12,13]. Effective breeding programs should therefore monitor population size, inbreeding risk, line identity and genetic diversity, especially when selected lines are maintained for many generations.

Field-performance validation is the decisive test of improvement. Inundative release of *T. dendrolimi* at different developmental stages and field performance of mass-reared *Psyttalia humilis* illustrate the importance of connecting laboratory indicators with practical release outcomes [38,71]. Release density, dispersal capacity and rearing conditions also influence the performance of *Telenomus podisi* and other egg parasitoids [81]. These examples show that improved traits must be evaluated in relation to release strategy, crop architecture, pest density and environmental conditions.

A recommended validation sequence for improved lines includes base population documentation; selection or improvement protocol; laboratory assay of the target trait; multi-trait fitness-cost evaluation; storage and transport test; comparison with the original line under semi-field conditions; field suppression test; and multi-generation stability assessment (Table 3). This sequence prevents overinterpretation of promising laboratory lines and makes it easier to identify which selected strains have practical value for biological-control programs.

## 7. Challenges and Future Directions

A major limitation in the current literature is that many studies still stop at screening rather than entering a true breeding design. Host suitability, artificial diets, storage conditions and strain differences are frequently compared, but relatively few studies establish replicated selection lines, report selection intensity, maintain control lines or evaluate selection responses across multiple generations. Without these elements, it is difficult to distinguish phenotypic screening from directional breeding. Future studies should therefore report breeding objectives, base population structure, number of generations, selection criteria, effective population size and statistical evidence for selection response.

Another important issue is the tendency to focus on single traits. Selection for pesticide resistance, cold tolerance, reduced flight ability or host killing can be valuable, but biological control depends on a combination of production, release and field-performance traits. A predator with high pesticide resistance but low reproduction may be commercially unattractive; a parasitoid with high host killing but poor emergence may be useful only in inundative release programs; and a flightless ladybird may remain longer in crops but fail to track pest patches. Future breeding programs should therefore adopt multi-trait evaluation indexes that combine production efficiency, release readiness and field efficacy.

The translation of laboratory performance into field performance remains a central challenge. Laboratory assays are essential for selection because they provide controlled and repeatable measurements, but they may overestimate performance in heterogeneous crop environments. Temperature fluctuation, crop architecture, pest density, refuge availability and interactions with other management practices can all alter the realized value of a selected trait. Field validation should therefore be incorporated earlier in the breeding cycle. Even small-scale field-cage or semi-field assays can help determine whether a selected trait leads to improved pest suppression, retention or persistence under more realistic conditions.

Genetic erosion also requires greater attention. Artificial selection and commercial production can narrow genetic variation, especially when colonies are maintained with small effective population sizes. Genetic monitoring should become a routine component of natural enemy improvement rather than an optional research add-on. Molecular markers, genome-wide SNPs, pedigree-free diversity metrics and periodic comparisons with source populations can help maintain line identity, detect bottlenecks and support long-term quality management [8,9,54].

The use of genetic and genomic tools must also be framed realistically. At present, the most feasible applications are marker-informed monitoring, candidate-gene discovery, transcriptomic interpretation and population-genomic quality management, rather than direct genetic engineering for commercial release. This distinction is important because it allows molecular tools to be integrated into biological-control industries while avoiding premature claims about genetically modified natural enemies. A cautious but practical approach will be more acceptable to regulators, producers and end users.

Future progress will depend on building trait databases for insect natural enemies, standardizing phenotyping protocols, linking phenotypic traits with genetic information, maintaining diverse germplasm resources and creating field-proven improved strains. The field should move beyond descriptive rearing optimization toward predictive, trait-oriented and genetically informed improvement of biological-control agents. Such a shift would make it possible to develop natural enemy lines that are not only easier to produce, but also more stable, more compatible with integrated pest management and more effective after release.

## 8. Conclusions

Natural enemies’ biocontrol efficacy and mass rearing are not merely technical steps in the production of insect natural enemies; they are evolutionary processes that can change the traits, genetic diversity and field performance of biological-control agents. A trait-oriented breeding perspective allows these processes to be managed more deliberately. Current evidence shows that insect natural enemies can be selected or improved for pesticide resistance, cold tolerance, starvation tolerance, diapause capacity for storage and release synchronization, nonreproductive host killing, zoophagy, pollen-feeding performance, body size, reduced flight ability and field retention. However, the same evidence also shows that trait improvement must be evaluated through trade-offs, genetic stability and field validation. The most promising future direction is an integrated improvement pipeline that combines conventional rearing-associated adaptation, artificial selection, experimental evolution, line crossing, phenotypic quality control, genetic monitoring and genomic resources. Such integration can help move biological control from mass production alone toward the development of stable, effective and field-proven improved strains of insect natural enemies.

## Figures and Tables

**Table 2:** Examples of selected traits and improved lines or strains of insect natural enemies for biological control.

Target Trait	Natural Enemy	Improvement Method	Key Limitation or Trade-Off to Evaluate	References
Cold tolerance	*Orius laevigatus*	Selective breeding of cold-tolerant lines	Effects on reproduction, predation and performance in warm conditions	[26]
Starvation resistance	*Pachycrepoideus vindemmiae*	Artificial selection	Potential correlated responses and field relevance	[27]
Zoophagy	*Dicyphus hesperus*	Artificial selection	Polyphagy/phytophagy and plant or fruit damage	[36]
Pesticide compatibility	*Orius laevigatus*	Selection for emamectin benzoate resistance	Trade-offs in fitness, predation, genetic stability and post-release persistence	[17,29,32]
Pollen-feeding performance	*Orius laevigatus*	Genetic improvement/selection	Context dependence under prey scarcity and plant species differences	[30]
Body size	*Orius laevigatus*	Selection for larger body size	Possible life-history trade-offs	[33]
Nonreproductive host killing	*Pachycrepoideus vindemmiae*	Artificial selection	Need to balance pest mortality with parasitoid reproduction	[31]
Reduced flight ability/flightless strain	*Harmonia axyridis*	Selective breeding and artificial selection	Possible survival, dispersal and ecological costs	[35]

**Table 3:** Suggested validation pipeline for selected or genetically improved insect natural enemy lines.

	Step	Objective	Recommended Reporting Items	Rationale
1	Base population definition	Document source of selectable variation	Geographic source, host/prey source, founder number, generation number	Allows evaluation of genetic background and reproducibility
2	Selection design	Distinguish selection response from drift	Target trait, selection intensity, generations, replicate lines, control lines	Prevents unsupported claims of improvement
3	Target-trait assay	Confirm improvement in the selected trait	Standardized assay protocol and comparison with control line	Provides direct evidence for trait response
4	Correlated-trait assay	Detect fitness costs or trade-offs	Fecundity, survival, sex ratio, dispersal, host/prey use, plant damage where relevant	Determines whether improvement is biologically useful
5	Storage and transport test	Evaluate commercial release readiness	Cold storage, handling survival, post-storage reproduction or predation/parasitism	Links selected trait to production logistics
6	Semi-field and field validation	Test pest suppression under realistic conditions	Release density, crop setting, pest density, retention, field efficacy	Confirms applied value of selected line
7	Genetic stability monitoring	Maintain quality across production cycles	Effective population size, inbreeding risk, molecular markers or genomic indicators	Prevents loss of quality during long-term production

## Data Availability

No new data were created or analyzed in this study. Data sharing is not applicable to this article.
